# A review on I–III–VI ternary quantum dots for fluorescence detection of heavy metals ions in water: optical properties, synthesis and application

**DOI:** 10.1039/d1ra08660j

**Published:** 2022-04-11

**Authors:** Bambesiwe M. May, Mokae F. Bambo, Seyed Saeid Hosseini, Unathi Sidwaba, Edward N. Nxumalo, Ajay K. Mishra

**Affiliations:** Institute for Nanotechnology and Water Sustainability (iNanoWS), College of Science, Engineering and Technology, University of South Africa, Florida Campus Johannesburg South Africa; Mintek Analytical Chemistry Division Private Bag X3015 Randburg 2125 South Africa; DSI/Mintek Nanotechnology Innovation Centre, Advanced Materials Division Private Bag X3015 Randburg 2125 South Africa; Department of Chemical Engineering, Vrije Universiteit Brussel Pleinlaan 2 1050 Brussels Belgium; Department of Medicine and Chemical Engineering, Hebei University of Science and Technology Shijiazhuang 050018 China ajaykmishraedu@gmail.com; Academy of Nanotechnology and Waste Water Innovations Johannesburg South Africa; Department of Chemistry, School of Applied Sciences, KIIT Deemed University Odisha India

## Abstract

Heavy metal contamination remains a major threat to the environment. Evaluating the concentrations of heavy metals in water environments is a crucial step towards a viable treatment strategy. Non-cadmium photo-luminescent I–III–VI ternary QDs have attracted increasing attention due to their low toxicity and extraordinary optical properties, which have made them popular in biological applications. Recently, ternary I–III–VI-QDs have gained growing interest as fluorescent detectors of heavy metal ions in water. Here, we review the research progress of ternary I–III–VI QDs for the fluorescence detection of heavy metal ions in water. First, we summarize the optical properties and synthesis methodologies of ternary I–III–VI QDs. Then, we present various detection mechanisms involved in the fluorescence detection of heavy metal ions, which are mostly attributed to direct interaction between these unique QDs and the metal ions, seen in the form of fluorescence quenching and fluorescence enhancement. We also display the potential applications in environmental remediation such as water treatment and associated challenges of I–III–VI QDs in the fluorescence detection of Cu^2+^ and other metal ions.

## Abstract 2

Ternary I–III–VI quantum dots used in the fluorescence detection of heavy metals ions in water.

## Graphical abstract

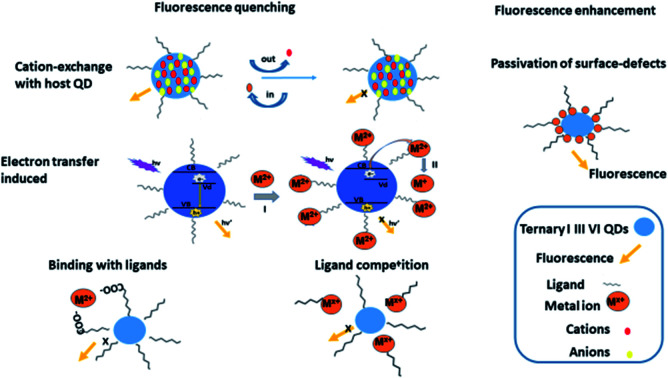

## 1. Introduction

Rapid economic development, urbanization, and population growth have increased the demand for resources for everyday operations, putting enormous pressure on natural and anthropogenic human activities such as industrial, agricultural, municipal, and mining activities to withstand this demand. Inevitably, this leads to more cases of environmental pollution especially water pollution due to the effluents that are inevitably generated during these activities.

The contamination of water systems by inorganic pollutants such as heavy metals remains a major concern. Copper (Cu), manganese (Mn), cobalt (Co) and zinc (Zn) are among the few heavy metals essential for human health, although they become toxic at high concentrations.^1^ Other metals like mercury (Hg), lead (Pb) and cadmium (Cd) are non-essential and toxic.^2,3^ These pollutants contaminate water streams which are used for irrigation purposes and agricultural land even at trace concentrations, posing a high health risk to the people consuming the water and agricultural products. Moreover, these pollutants are not biodegradable therefore they are persistent in water streams and agricultural land. The prolonged exposure of humans to these metals can cause various diseases, *e.g.* brain damage, kidney dysfunction, organ failure, and bone disorder.^4,5^ Hence, the World Health Organisation (WHO) and the United States Environmental Protection Agency (USEPA) have established maximum concentration levels for some of these metals of concern.^6^ Thus, it is imperative to detect these pollutants to protect the environment and prevent diseases. Additionally, the proper evaluation of the amounts of heavy metals present in the water environments is a crucial step towards the formation of a viable treatment plan.

The analysis of heavy metal ions in water requires highly sensitive and selective technologies that can easily integrate with microfluidic media. Atomic absorption spectroscopy (AAS), inductively coupled plasma optical emission spectroscopy (ICP-OES), and mass spectroscopy (ICP-MS) are among the most common analytical techniques for the determination of heavy metals.^7,8^ They show excellent sensitivity, and some are with multi-element analysis capability (*i.e.* ICP-OES and ICP-MS). However, they are expensive and often involve sample preparation methods which can be complex, time-consuming, and error prone.

Nano-based material sensing has emerged as a key role player in the field of heavy metal ion detection.^9–16^ Since these materials are in the nanoscale, their unique chemical and physical properties such as large surface area, chemical affinity, and surface activity can aid the sensing processes.^17,18^ Among the nano-sensing systems, quantum dot (QD)-based fluorescent probes have gained popularity due to their efficient small catalytic sizes which help to display low detection limits, fast response time, high specificity,^19–21^ anti-pathogenic effects (*e.g.* virucidal^22^ and bactericidal properties^23,111^) and high influx membrane-based antifouling metal-doping effects.^24,110^ Also, they exhibit optical properties such as high photoluminescence quantum yield (PLQY), photoluminescence (PL) spectra from the ultraviolet-visible to the near-infrared and high photostability, which make them ideal fluorescence sensors^25^ as well as photodegradation, and photodynamic therapeutic agents.^23^ As sensors, their detection strategy is based on compromising or enhancing their fluorescence properties upon exposure to the analyte, which can be seen with changes in intensity, PL lifetime, and emission peak position at varying concentrations of the analyte.^26^

In the past years, QD nanomaterials have been employed for water treatment purposes in the form of sensors (for inorganic and organic targets), photocatalysts^27^ and microbial inhibitory agents.^23^ Among these nanomaterials, several II–VI based (CdSe, CdS) QDs^28,29,117,118^ and ZnS QD based fluorescent sensors^30,31^ were designed for screening of heavy metal ions. However, ternary I–III–VI QD probes are still the least explored in this field. Ternary I–III–VI QDs have been receiving growing interest mainly because of their attractive optical and electronic properties and offering of safer alternatives compared to II–VI and IV–VI (PbSe) QDs.^32–34^ This makes them popular in biological applications,^35–39^ water treatments and biocompatible solar cells applications.^37,40,41^ Specifically, this safety advantage has been extended by demonstrating the analytical potential of I–III–VI QDs as heavy metal ion detectors in water.^42–44^ The comprehensive review on the biological,^35,36^ photocatalysis/disinfections^23^ and energy^45^ applications of QD materials have been previously reported by several authors and thus will not be covered in this review. Thus, this review aims at presenting the research progress on I–III–VI ternary QDs as fluorescent detectors for heavy metal ions in water. The detection mechanisms based on the direct interaction between the QDs and metal ions, evident by fluorescence quenching and fluorescence enhancement are fully described. Also, the potential in real water treatment applications together with challenges and future prospects of these materials is fully elucidated.

## 2. Optical properties of I–III–VI QDs

Quantum dots (QDs) have become quite popular over the years due to their striking optical and structural properties.^29,37,45^ Particularly, I–III–VI ternary QDs (*e.g.* CuInS_2_, CuInSe_2_, AgInSe_2_, and AgInTe_2_) have emerged as a new class of biological labels because they exhibit longer fluorescence lifetimes compared to binary QDs and long excited-state lifetimes, which makes them excellent fluorophores in biological applications.^36,37,46^ In the early years, I–III–VI ternary QDs have been mainly studied for their high potential in solar energy conversion, light-emitting displays and thin layer photovoltaics since they offer tuned emission from the visible to near-infrared (NIR) region.^47–52^

I–III–VI ternary QDs are derived from II–VI binary QDs by replacing the divalent cation with one monovalent and one trivalent cation to synthesize less toxic QDs with similar excellent optical properties. Unlike II–VI and IV–VI binary QDs, most ternary QDs do not exhibit a sharp exciton peak but rather an absorption band edge. This is a known feature of ternary QDs, which is associated with surface defects on the crystal structure, making it a challenge to determine the size-dependent bandgap.^53^ Considering this challenge, Mao *et al.*^47^ demonstrated a theoretical calculation of the I–III–VI band gap using Tauc law from the absorption band edge of the semiconductor material.

1 (*Ahν*) *α*(*hν* − *E*_g_) 1/2 for *hν* < *E*_g_

In the equation, *hν* is the energy of the absorbed photons, *E*_g_ is the bandgap of the semiconductor material and *A* is the absorption band edge.^47^

### 2.1 Photoluminescence mechanism of I–III–VI QDs

In principle, when QDs are excited, an electron is promoted from the valence band to the conduction band, leaving a hole. This is followed by two pathways; (i) the recombination of the electron and hole in the valence band to form excitons. These excitons can either undergo radiative decay which results in emission or non-radiative decay which results in heat release, (ii) the electron can be trapped by surface defects, which can back transfer and reproduce excitons which can also either undergo radiative decay or non-radiative decay.^29,54^

**Fig. 1:**
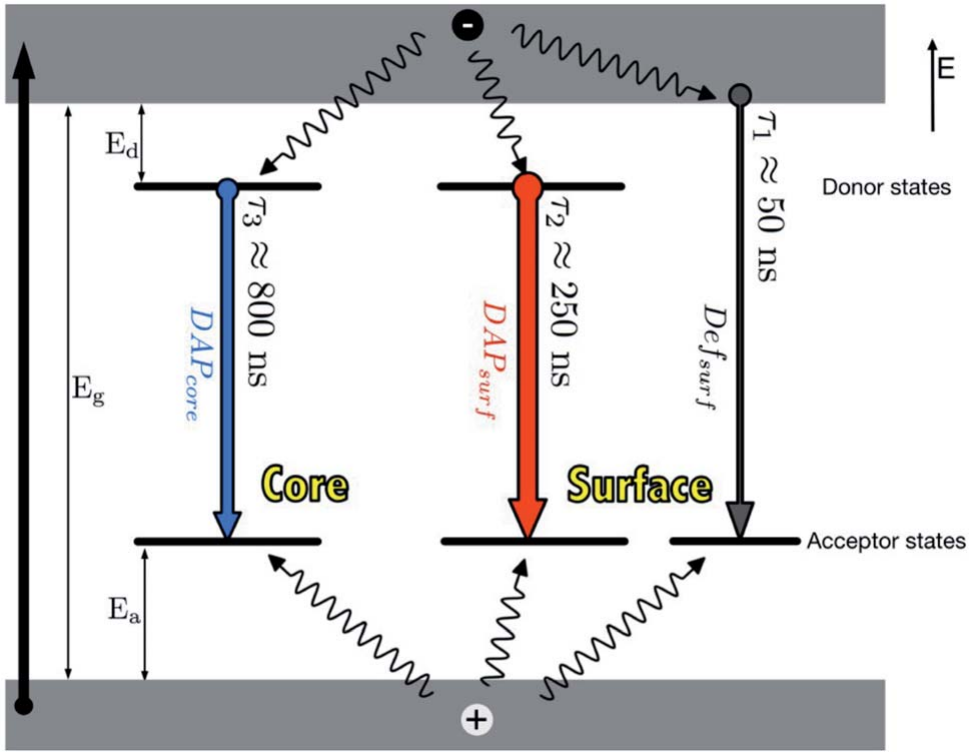
Photoluminescence mechanisms in AgInS_2_/ZnS QDs [reprinted with permission from ref. 55 Royal Society of Chemistry].

The photoluminescence (PL) characteristic features of I–III–VI ternary QDs include broad full width at half maximum (FWHM) (*i.e.* between 80–120 nm) of the PL peak, large stokes shift and slow PL lifetimes compared to binary QDs.^35,37,40,53^ These features mainly arise due to their various defects. For example, in CuInS_2_ core QDs defects such as Cu vacancies (V_Cu_), indium–copper (In–Cu) antisite defects and sulfur (S) vacancy defect may occur. Additionally, the varied molecularity and mole ratios in Cu, In and S precursors involved in the synthesis are also contributors to these features.^40^ Time-resolved PL (TRPL) spectra are often measured to recognize the PL lifetimes in the recombination process during emission. TRPL of ternary I–III–VI QDs reveals multiple emission states (Fig. 1) which arise from surface-related states due to dangling bonds, donor–acceptor pairs (DAP) involving surface defects (DAP_surf_), or intrinsic defects (DAP_int_).^55,56^ Furthermore, some ternary I–III–VI QDs revealed DAP_surf_ and DAP_int_ as the dominating components contributing towards their emission.^43,56,57^

### 2.2 Photoluminescence quantum yield

The core QDs usually exhibit poor photoluminescence quantum yield (PLQY) and photostability. This is because their small particle size permits a large surface area that consists of lots of atoms. Thus, even the least disturbance on the surface can lead to surface defects causing a noticeable change in the PL properties. Therefore, a material with a larger bandgap such as zinc sulfide (ZnS) can be used to passivate the surface to form a core/shell structure. This increases the PLQY, PL lifetime and stability of the QD, which is an indication of suppressed surface defects.^35,58–60^ Although ZnS shell coating has proven to increase PL properties, there are reports on an unexpected blue shift of the PL peak during the growth of the ZnS shell.^61–64^ A few propositions such as surface reconstruction,^65^ etching of the plain core material,^62^ inter-diffusion of Zn atoms^61^ and cation exchange^63^ have been suggested.

In another case, Mao *et al.*^53^ investigated the PL behavior of AgInS_2_ core and AgInS_2_/ZnS core/shell QDs. The results showed the reduction of surface defects with an increase in the particle size of the core QDs and further reduction of surface defects after ZnS shell coating. The PL spectra showed two deconvolution peaks relative to the QD emission peak, where peak 1 (in shorter wavelengths) was attributed to surface defects and peak 2 (at longer wavelengths) was attributed to intrinsic trap states. Intrinsic trap states are known to easily form in I–III–VI compounds between interstitial atoms which are responsible for donor–acceptor transitions. The results revealed a decrease in the emission intensity at peak 1 and an increase in peak 2 when the particle size increased with time. The same result was observed after the ZnS coating, which leads to higher PLQY. The reduced surface defects at an increased size are attributed to the fact that larger particles exhibit reduced surface to volume ratio which leads to fewer surface defects.^53^

In other reports, Li *et al.*^62^ enhanced the poor photoluminescence properties of CuInS_2_ core QDs by coating with cadmium sulfide (CdS) shell. An initial blue-shift followed by a red-shift in PL peak of CuInS_2_ core QDs with continuous CdS shell growth was reported. This was attributed to the lower conduction band of CdS compared to ZnS. PLQY increased from 5–10% to 86% after the growth of CdS shell and PL lifetime studies revealed that the non-radiative rates were largely reduced after coating of the core QDs with the shell.^62^ The coating of silica on CuInS_2_/ZnS core/shell QDs revealed enhanced stability but the PLQY was slightly reduced.^66^ Furthermore, stability was maintained when CuInS_2_/ZnS QDs were coated with ZnGa_2_O_4_.^67^ Rao *et al.*^68^ presented the self-passivation of Al_2_O_3_ on CuInS_2_/ZnS QDs to improve photostability. The Al_2_O_3_ passivation prevented the oxidation of sulfur on the surface of the QD after irradiation, suggesting that it improved photostability of the CuInS_2_/ZnS core/shell QD.^68^

Over the years, doping of QDs has been extensively explored.^58,69–72^ Doping is a process that involves introducing atoms to QDs to achieve additional properties. For instance, Guo *et al.*^73^ reported enhanced PLQY when Zn^2+^ was incorporated into CuInS_2_ core QDs to form quaternary CuInZnS QDs. The author also demonstrated the inhibition of the emission blue-shift during ZnS shell growth on CuInS_2_ QDs when Zn ions were intentionally introduced into the CuInS_2_ core QDs. This action inhibited the cation-exchange process occurring between Zn and the cations (Cu^2+^/In^3+^) during ZnS shell formation.^73^ In other reports, doping I–III–VI ternary QDs with Mn^2+^ or Gd^3+^ introduced a magnetic property which resulted in negligible decreases in PLQY and their application in fluorescence/magnetic resonance imaging studies.^70,74,75^

### 2.3 Photoluminescence lifetimes

The chemistry of core/shell structures of I–III–VI ternary QDs is complex and the effect of Zn diffusion on their photophysical properties is still under debate. Besides the PLQY increase, Zn diffusion can also alter the size of the QD which could also result in increased PL lifetimes. This means increased PL lifetimes are not solely attributed to reduced surface defects but could also be attributed to change in size. For instance, Komarala *et al.*^60^ reported the synthesis of AgInS_2_ core and AgInS_2_/ZnS core/shell QDs of different sizes by varying reaction time and temperature. The AgInS_2_/ZnS QDs emission was positioned at 600, 650, and 760 nm with PL lifetimes corresponding to 94 ns, 125 ns, and 200 ns respectively; indicating increased PL lifetimes at increased sizes. In the same study, the PL lifetime of AgInS_2_ core QDs increased from 68 ns to 200 ns after ZnS passivation (AgInS_2_/ZnS emitting at 760 nm) while surface-defect decay component was reduced from 61% to 36% and intrinsic defect decay component increased from 39% to 64%, indicating suppressed surface defects and induced intrinsic defects.^60^

On the other hand, Sharma *et al.*^56^ demonstrated the effect of increasing Zn quantities on AgInZnS QDs of the same size to separate the size effect from the composition effect. The study showed a gradual decrease in the PL lifetime at increased Zn amounts, while the surface defects decay components increased from 22 to 57% at increased Zn amounts, which was an opposite result to Komarala's report.^60^ In addition, the slow decay component was reduced from 21 to 3% at increased Zn amounts, an indication that the surface-related traps involving dangling bonds were reduced. The study also gave insight into the changes in non-radiative (*k*_nr_) and radiative recombination (*k*_rad_) rates at varied PLQY. The PLQY of AgInZnS nanocrystals (NCs) increased with increased Zn amounts (*x* = 1 to *x* = 0.7), while higher Zn amounts (*x* = 0.3) reduced the PLQY. The average PL lifetime (*τ*), and PLQY values of the NCs were used to determine the non-radiative (*k*_nr_) and radiative (*k*_rad_) recombination rates (Table 1) using equations below.^56^2
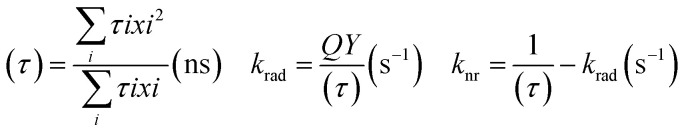


**Table 1:** PL quantum yield (PLQY), average PL lifetime (*τ*), decay component contribution (*x*), radiative decay rate (*k*_rad_) and non-radiative decay rate (*k*_nr_) for different compositions of AgInZnS at 3.4 eV excitation.^56^

AgInZnS(*x*)	PLQY (%)	*τ* (ns)	*k*_rad_ (s^−1^)	*k*_nr_ (s^−1^)
1	40.4	2518	0.16 × 10^6^	0.24 × 10^6^
0.7	70.6	1647	0.43 × 10^6^	0.18 × 10^6^
0.3	53.0	1017	0.52 × 10^6^	0.46 × 10^6^

The results showed that Zn initially increased PL lifetime where non-radiative surface decays were reduced and the radiative sites within the NCs were increased, then higher Zn quantities increased the non-radiative surface decays.^56^

## 3. Synthesis of I–III–VI ternary QDs

Generally, synthesis of I–III–VI ternary QDs usually requires thiol (S–H) ligands such as dodecanethiol (DDT), glutathione (GSH), thioglycolic acid (TGA), which serve as stabilizers. The thiol atoms are necessary for binding the ligand chain to the QDs surface *via* the sulfur bond. Furthermore, these materials are challenging to synthesize compared to binary QDs mainly because of the unbalanced reactivity of the cations. The cation in group I (*e.g.* Cu^+^, Ag^+^) is a soft acid while the other in group III (*e.g.* In^3+^) is a hard acid. Since the anion (Se^2−/^S^2−^) is a soft base, the cation in group I can react faster and form a stronger bond with Se^2−/^S^2−^ than group III cation. This makes it imperative to implement appropriate ligands to avoid phase separation and generation of binary material which are not of interest.^59^ Several authors addressed the challenge by using (i) single-source precursors,^76^ (ii) carboxylic acid and thiol ligands to control the reactivity of In and Cu cations respectively,^77^ and (iii) excess thiol ligand.^78^ Another issue is the possible formation of multiple peaks in the PL spectra. These were observed in AgInSe_2_ core QDs and might have originated from structural defects and poor crystallinity.^79^ However, synthesis parameters such as cation and anion mole ratio, ligands, pH, temperature, and reaction time can be varied to produce the desired material.^76^ The desired purpose of the QDs determines the type of synthetic approach to be employed for their preparation. These approaches can be broadly categorized as organic and aqueous syntheses.

### 3.1 Organic synthesis

Organic synthesis approaches produce high-quality I–III–VI ternary QDs and are adopted from the organic synthesis of binary QDs. These approaches include hot injection, thermolysis and solvothermal and usually involve metal complexes, cation/anion precursors and hydrophilic ligands dissolved in organic solvents (*e.g.* octadecene (ODE)) at high temperatures.^35,38,62,63,76,80–82^ Due to the sensitivity of the reagents to oxygen or air, an inert atmosphere is often required. For instance, Malik *et al.*^80^ were one of the first to demonstrate the synthesis of highly crystalline CuInSe_2_ nanoparticles through a hot injection method. Here, Cu and In salts were dissolved in trioctylphosphine (TOP), injected in hot trioctylphosphine oxide (TOPO) solution (previous degassed with N_2_ gas) and followed by addition of TOP(Se).^80^

Although organic synthesis produces high-quality QDs, it might not be suitable for all applications. For example, biological applications require the material to be biocompatible, hydrophilic, and non-toxic. Therefore, it is imperative to change the organically soluble QDs to water-soluble without losing their optical properties. This can be achieved through ligand–exchange with hydrophilic ligands, silica shell coating as well as encapsulation with amphiphilic ligands. A desirable ligand of interest is usually thiols with water-soluble functional groups such as carboxylic groups. Thiol binds to the surface of the QDs and the water-soluble groups on the surface make it soluble in aqueous media. An example of such ligands is 11-mercaptoundecanoic acid (MUA), which was reported to be biocompatible and low in toxicity.^46^ Mao *et al.*^46^ reported ligand exchange of DDT-stabilized AgInS_2_/ZnS QDs with MUA ligand. The process involved the dispersion of QDs with MUA in chloroform under sonification. The absorption and PL spectrums were like those obtained for DDT-stabilized QDs with emission peaks positioned at 809 nm. A time-resolve study was carried out, where biexponential function showed a decrease for both *r*_1_ (fast decay) and *r*_2_ (slow decay) components which led to a decrease in the PL decay average (*r*_ave_) from 349.4 to 324 ns. This was attributed to the decreased stability of the QD surface due to the exchange of the ligands. Regardless, the *r*_ave_ value was still higher than that obtained for II–IV binary QDs.^46^ Although water-soluble QDs can be produced through ligand–exchange, a decrease of 5–35% in PLQY usually occurs.^78,83,84^ This decrease has been attributed to the surface traps due to poorly coated QD surface after passivation. Besides, the reactions involve long reaction times and then transfer to water-soluble means additional synthesis steps which can be tedious. Nonetheless, the synthesis of organic soluble QDs requires high temperatures, large quantities of organic solvent, and sometimes make use of unstable and hazardous precursors which are not ideal for green synthesis. Therefore, a lot of attention has been paid to direct aqueous synthesis of ternary QDs.

### 3.2 Aqueous synthesis

Aqueous synthesis approaches are cheaper, simpler, and much greener in nature. Liu *et al.*^78^ demonstrated the hydrothermal synthesis of CuInS_2_ QDs with a molar ratio of 1 : 1 : 12 for Cu : In : mercaptopropionic acid (MPA) where the excess MPA played a role in balancing the cation reactivities. The reaction was carried out at 150 °C in an ambient atmosphere for 21 h. The study also explored the use of other thiol stabilizers such as 2-mercaptonicotinic acid (MNA) and mercaptosuccinic acid (MSA). The results showed a blue-shifted emission peak for MSA and no PL emission for MNA as the bulk material was precipitated.^78^ Unlike most organic synthesis where DDT balances the cation reactivity, the imbalanced reactivity of group I and III cations in aqueous synthesis is more critical, thus the use of dual stabilizers. Chen *et al.*^88^ reported the synthesis of water-soluble CuInS_2_/ZnS core/shell QDs, where GSH and sodium citrate were used as dual stabilizers. The experiment was conducted at low temperatures of 95 °C in the absence of an inert atmosphere. Na_2_S was used as the sulfur source in the CuInS_2_ core QD synthesis, which is highly reactive at low temperatures and essential for small-sized CuInS_2_ QDs. Thiourea was used as a sulfur source during the ZnS shell growth. The emission peaks were tuneable from 543 to 625 nm by altering the copper content. The PL intensity of the QDs increased with the growth of ZnS shell on CuInS_2_ cores with PLQY up to 38%. The biexponential decays and average fluorescence lifetimes were similar to those previously reported,^85–87^ revealing success in reducing surface defect recombination sites.^88^

## 4. Fluorescence detection mechanisms

**Fig. 2:**
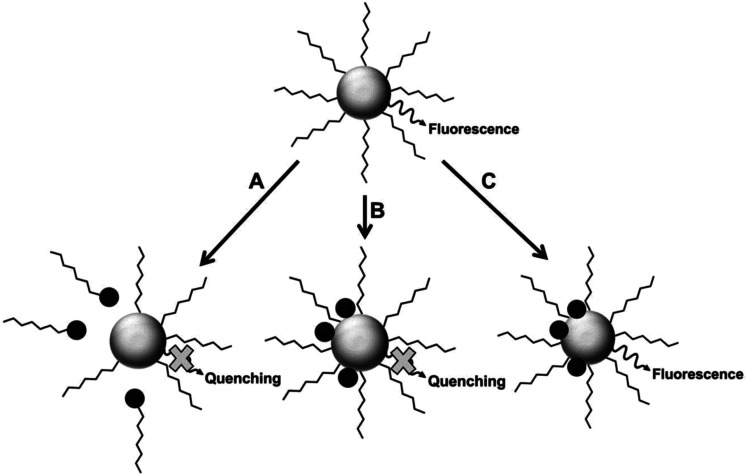
Proposed mechanisms for analyte interaction with the NP samples [reprinted with permission from ref. 89 American Chemical Society].

Fluorescence intensity can be used to determine the concentration of a fluorescent species, in addition to detecting the presence of an analyte in a medium. The latter can be achieved *via* fluorescence quenching or enhancement after interaction of the analyte with the fluorophore.^26^ Some of the fluorescence detection mechanisms are shown in Fig. 2.

Fluorescence mechanism A assumes the attack of surface ligand by a metal ion, resulting in detachment of the ligand. This destabilizes the QD, leading to reduced fluorescence.^90^ Mechanism B suggests direct interaction with the host QD, which could result in cation exchange with the metals in the QD.^42,43,89,91^ Mechanism C involves forming passivating layers with QDs surface, which cover surface defects and enhance fluorescence.^44,89,92,93^

### 4.1 Fluorescence quenching

Fluorescence quenching refers to the process of reducing fluorescence properties of a fluorophore, where the photoluminescence peak intensity decreases. Quenching that results from collisional and static encounters is referred to as dynamic (collisional) and static quenching, respectively.^94^ In both cases, the quencher and the fluorophore must be in contact. Dynamic quenching involves the binding of the quencher onto the fluorophore, which can be short-lived and omit the emission of a photon. In static quenching, the binding could form a non-fluorescent complex between the fluorophore and the quencher.^95–98^ Investigating the type of quenching can give one valuable information about the type of binding between the fluorophore and the quenching species.^94^ Quenching mechanisms can be attributed to several pathways such as competition of surface ligands,^29,90^ binding with surface ligands,^99,100^ and cation exchange with the host QDs.^42,43,89,91^

Fluorescence quenching can also be a result of the inner filter effect (IFE) which occurs when a species absorbs excited/emitted light during the detection process. This can be seen in the overlap of the absorption spectra of the absorber and the excitation spectra of the fluorophore.^21^ IFE is not categorized as a major quenching process since it does not involve radiative and non-radiative transitions in fluorescence measurements.^101^ Therefore, other mechanisms besides IFE could be attributed to the quenching. Moreover, IFE has been reported to enhance the sensitivity compared to other mechanisms because the changes in the absorbance of sensors can transform exponentially into fluorescence intensity changes.^102–104^

#### 4.1.1 Static quenching: inner filter effect

**Fig. 3:**
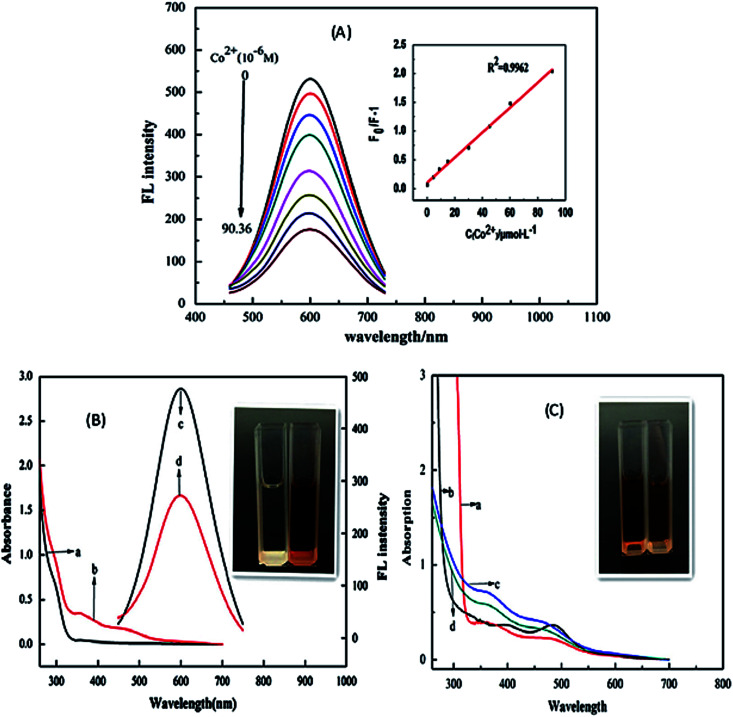
(A) PL intensity of the CuInS_2_/ZnS/TGA QDs in addition of various concentration of Co^2+^. Inset: The extent of the quenching (*F*_0_/*F*_I_) *versus* the concentration of Co^2+^. (B) UV-vis absorption spectra (a and b) and PL spectra (c and d) of QDs solution in the absence (a and c) and presence (b and d) of Co^2+^. Inset: the photo of QDs solutions in the absence (left) and presence (right) of Co^2+^ in natural light. (C) UV-vis absorption spectra of mixture of Co^2+^ and TGA (a), mixture of Co^2+^ and thioglycerol (b), and mixture of QDs and Co^2+^ in the absence (c) and presence (d) of excess carboxyl. Inset: The photo of the mixture solutions of TGA (left) and thioglycerol (right) with Co^2+^ respectively in natural light [reprinted with permission from ref. 90 copyright 2013 Elsevier].

**Fig. 4:**
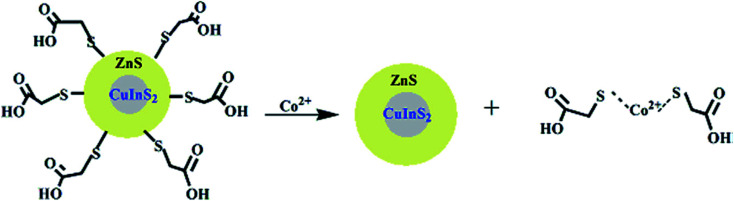
Schematic representation for the coordination of Co^2+^ ions and CuInS_2_/ZnS/TGA QDs [reprinted with permission from ref. 90 copyright 2013 Elsevier].

Since static quenching is associated with complex formation between the fluorophore and the quencher. It could be linked to the competition of ligands between the QDs and metal ions, which could lead to ligand–metal complex formation. Zi *et al.*^90^ demonstrated the use of TGA-capped CuInS_2_/ZnS QDs for the fluorescence detection of Co^2+^ ions in water. The PL peak intensity was gradually reduced upon the addition of Co^2+^ ions at increased concentrations, accompanied by a drastic color change of the QD solution (Fig. 3A and B). The quenching was attributed to the formation of a TGA–(Co^2+^)–TGA complex; where the Co^2+^ ion bonded to the TGA molecule through sulfur resulting in the detachment of the TGA molecule from the QD resulting in a surface deficient QD (Fig. 4). This induced non-radiative recombinations resulting in a destabilized nanoparticle, hence the decrease in the PL intensity.^90^

Anand *et al.*^105^ and Gore *et al.*^106^ speculated TGA–metal complexes were either due to interaction between the metal ion and the carbonyl group or sulfur group respectively.^105,106^ Therefore, absorbance spectra of Co^2+^ ions exposed to TGA and Co^2+^ ions exposed to thioglycerol were investigated to determine the interaction. The results showed two peaks centered at 370 nm and 470 nm for Co^2+^–TGA solution and similar spectra with slight shifts were observed for Co^2+^–thioglycerol solution, which was attributed to the presence of carbonyl groups in the TGA solution. The similar spectra indicated that the interaction between Co^2+^ and TGA was indeed through the sulfur group and not the carbonyl group (Fig. 3C).

The effect of temperature on the fluorescence intensity of the QDs with Co^2+^ ions was studied to confirm the type of quenching. During dynamic quenching, the quenching constant increased while in static quenching, the constant decreased with a rise in temperature. The data was analyzed using the Stern–Volmer equation and the results revealed an inversely proportional relationship with temperature, confirming static quenching mechanism.^90^

Establishing the type of quenching gave insight into the binding involved in the quenching. Thus, IFE was identified as the partial contributor towards the quenching of the QDs upon exposure to Co^2+^ ions. This could be confirmed by the overlap in the excitation wavelength of the QDs at 365 nm with the absorption spectra of the QDs-Co^2+^ solution (Fig. 3B). This indicated that less light entered the QDs when excited at 365 nm, thus reducing the fluorescence because changes in absorbance could exponentially transfer to the resulting fluorescence properties.^90^

#### 4.1.2 Binding with surface ligands

**Fig. 5:**
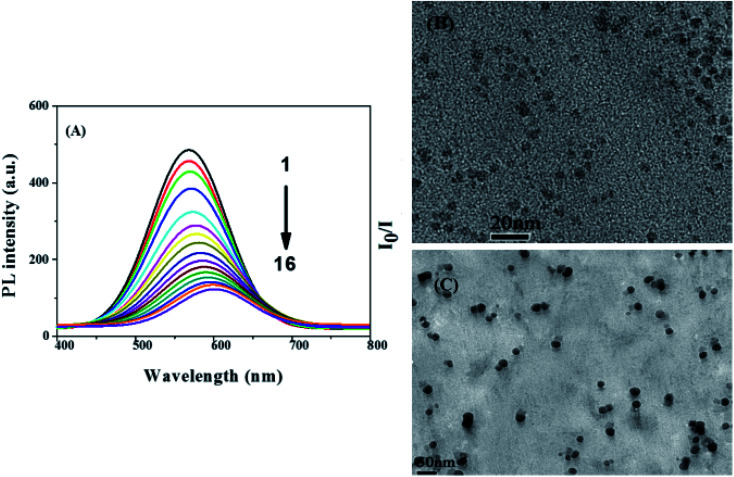
(A) The fluorescence spectra of AgInZnS–graphene oxide (GO) nanocomposite after addition of different Cu^2+^ concentrations of sample 1 to 16 *i.e.* 0, 10, 30, 60, 100, 150, 200, 250, 300, 350, 400, 500, 550, 650, 750, 850 μM, respectively. (B) TEM images of AgInZnS–GO NC in absence and (C) presence of Cu^2+^ ions (850 μM) [reprinted with permission from ref. 99 copyright 2016 Elsevier].

The binding of metal ions onto surface ligands occurs through electrostatic interaction between the metal cations and the negatively charged QD surface and since metal ions are electron deficient, electrons may be transferred from QD to the metal ion. This interaction is common with QDs that exhibit highly dense ligands or functionality on their surface, preventing the attack on the core material.^29^ Lui *et al.*^99^ displayed the AgInZnS–graphene oxide (GO) nanocomposite (NC) fluorescent probe for Cu^2+^ ion detection. The PL quenching of the NC upon addition of Cu^2+^ was attributed to the reaction between the carbonyl group on the NC surface and the Cu^2+^ ion forming a carboxylate Cu complex (R–COO–Cu–OOC–R). This induced defects which lead to quenching. Furthermore, the fluorescence peak gradually red-shifted with increased Cu^2+^ ion concentration, suggesting increasing particle size (Fig. 5(A)). This was also supported by TEM images, which demonstrated a particle size increase from 5.7 nm to 21.7 nm in the absence and presence of Cu^2+^ ion respectively (Fig. 5B and C).^99^ Another report displayed PL quenching of AgInS_2_ QDs after they were exposed to Pb^2+^ ions.^57^ The QDs were capped with MSA, a dicarboxylic acid with a thiol group. It is suspected that the Pb^2+^ ions bonded to the carbonyl groups on the QD surface to form R–COO–Pb^2+^–OOC–R complex as seen in Lui *et al.* report,^99^ which might have induced defects, thus increasing non-radiative decay pathways.^57^

**Fig. 6:**
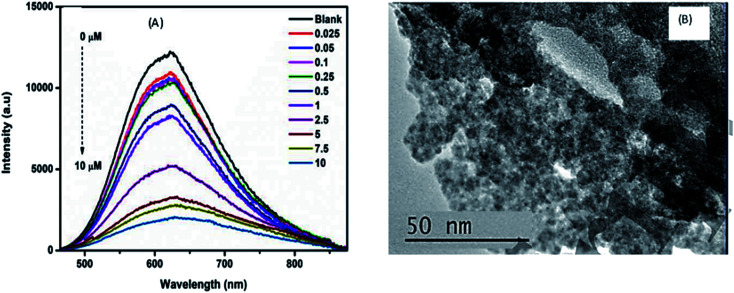
(A) The PL spectra of GSH capped AgInS_2_–ZnS QDs after addition of different Cr^3+^ concentrations. (B) TEM images of GSH capped AgInS_2_–ZnS QDs after addition of Cr^3+^ ions [reprinted with permission from ref. 100 IOP Publishing].

Parani *et al.*^100^ reported GSH capped AgInS_2_–ZnS QDs for the detection of Cr(iii) ions. The PL intensity of the QDs was quenched with increased Cr^3+^ ion concentration between 0.025 μM and 10 μM, accompanied with gradual red-shifting of the PL peak from 623 nm to 630 nm (Fig. 6A). The quenching behaviour was attributed to the aggregation of the QD after addition of Cr^3+^ ions, seen in the TEM image (Fig. 6B). The authors proposed that the aggregation was due to the attachment of Cr^3+^ ion onto the surface GSH; since Cr^3+^ is a hard acid, it binds to oxygen and nitrogen rich ligands, GSH being rich in these, a Cr^3+^–GSH complex forms on the surface. FTIR analysis showed significant shifting of the COO^−^ peaks to higher wavenumbers after addition of Cr^3+^ ions, confirming that the Cr^3+−^GSH interaction occurred through the GSH carboxylate group likely by electrostatic interaction. Furthermore, the Cr^3+^–GSH complex formation resulted in reduced electrostatic repulsion between the QDs hence the aggregation. As a result, an imperfect QD surface was initiated which facilitated non-radiative recombination hence the quenched PL^100^^.^

#### 4.1.3 Electron transfer induced quenching

**Scheme 1:**
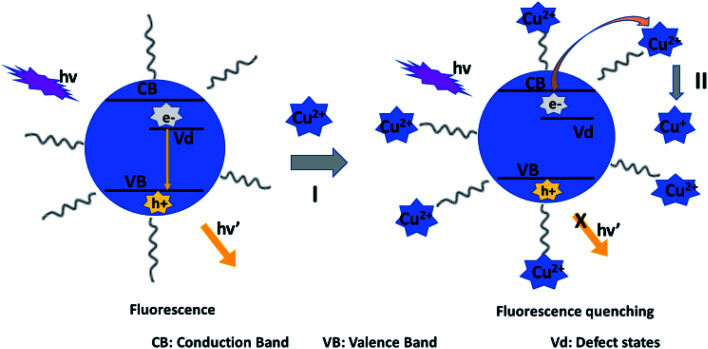
Schematic illustration for the fluorescence quenching of DTAB-capped AIZS QDs by Cu^2+^ ions [reprinted with permission from ref. 107 copyright 2017 Elsevier].

Here, the electrons are directly transferred from the QD to the metal ion, then the metal ion is reduced forming non-radiative surface channels. This PL quenching is common with the detection of Cu^2+^ ion, where Cu^2+^ is reduced to Cu^+^.^29^ For example, dodecyltrimethylammoniumbromide (DTAB) capped AgInZnS QDS displayed fluorescence quenching after addition of Cu^2+^ ions. X-ray photoelectron spectroscopy (XPS) analysis of the QDs in the presence of Cu^2+^ ions revealed the presence of Cu (2p) peak corresponding to Cu^+^ in QDs. These results suggested the PL quenching mechanism might be attributed to electron transfer from the QD to Cu^2+^ ions, resulting in the reduction of Cu^2+^ to Cu^+^. This occurs when electrons attempt to recombine with holes in the (valence band) *V*_B_, but get trapped to defect traps, where some electrons are transferred to Cu^2+^ ion thus inducing non-radiative pathways resulting, in some extent, to PL quenching (Scheme 1).^107^

The authors also displayed this mechanism in sodium dodecylsulfate (SDS)-capped AgInZnS QDs. The zeta potential of the QDs was −36.8 mV, demonstrating that the negatively charged QDs bonded with Cu^2+^ through electrostatic interactions which resulted in the electron transfer from the QD to the Cu^2+^. This aided the reduction of Cu^2+^ to Cu^+^, which was also confirmed by XPS analysis.^108^

Later, an optical fiber nanoprobe based on (SDS)-capped AgInZnS QDs was developed for the detection of Cu^2+^ ions. The QDs were deposited on silica optical fiber ends using polyvinylalcohol (PVA) as an entrapment matrix. The PL intensity of the QDs was reduced after the addition of Cu^2+^ ions, while the average lifetime of the QDs decreased from 500 ns to 325 ns. The reduced PL decay could be due to the reduced radiative pathways facilitated by some electrons being transferred to Cu^2+^ ions.^109^

**Scheme 2:**
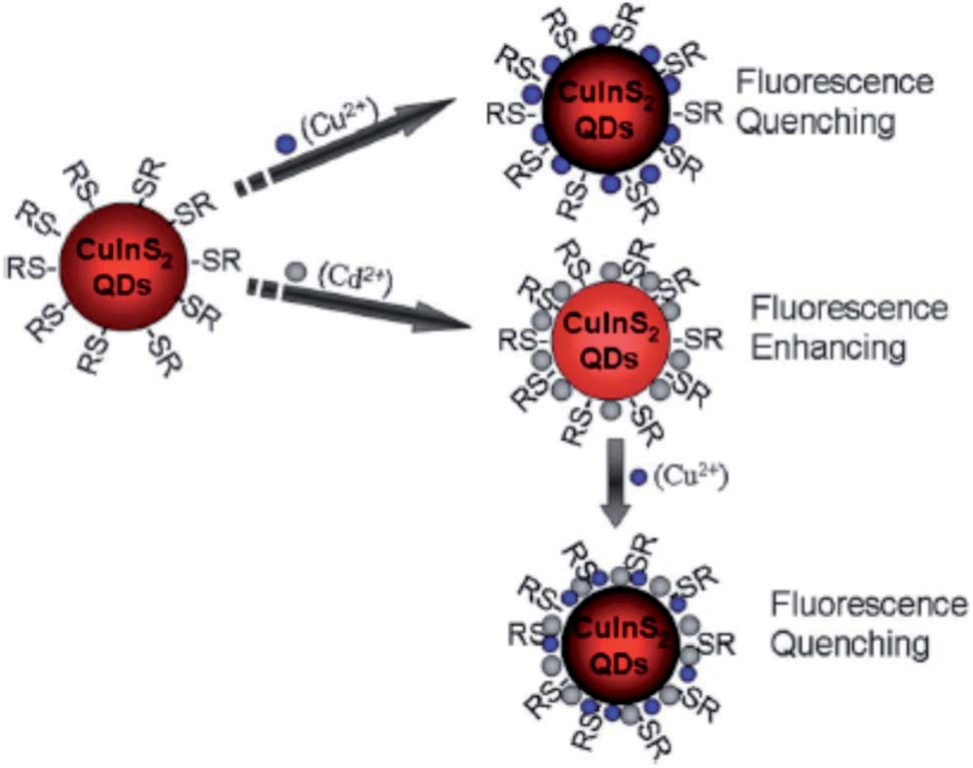
Cu^2+^ and Cd^2+^ ion detection process using CuInS_2_ QDs [reprinted with permission from ref. 44 Royal Society of Chemistry].

Liu *et al.*^44^ reported fluorescence detection of Cu^2+^ ions using MPA-capped CuInS_2_ QDs. The PL quenching of the QDs upon exposure to Cu^2+^ was attributed to the formation of either Cu_2_S precipitate or isolated Cu^+^ ions on the surface of the MPA capped QDs. This was facilitated by the reduction of Cu^2+^ to Cu^+^ through electron transfer from QDs to Cu^2+^ ions. In addition, the QD–Cu^2+^ mixture was exposed with EDTA, a Cu^2+^ extracting agent. No changes in the PL spectra was observed, indicating that the quenching reaction could not be reversed, suggesting that static quenching could also be involved. Modifying the QDs with Cd^2+^ played a profound role in improving the sensitivity, detection range and limit. In this reaction, the quenching might be due to the Cu^2+^ adsorption on the surface of Cd modified QDs, which changed the original surface state (CuInS_2_–Cd-SR), inducing the non-radiative recombinations and thus reducing the radiative emission (Scheme 2).^44^

#### 4.1.4 Cation exchange with host QDs

Cation-exchange is the replacement of cations in an ionic crystal with guest cations while maintaining the original anionic structure and rarely changing the size.^105–107^ It is an emerging strategy in II–VI, IV–VI, and I–III–VI type QDs for heavy metal sensing applications.^31,42,91,113^ For example, II–VI QDs (*e.g.* CdS) are formed by a combination of cations and chalcogenide anions. The cation in the QD can be displaced and exchanged with heavy metal cations (*e.g.* Hg^2+^) since the *K*_sp_ values of the resulting crystal (HgS) are higher than CdS.^114^ Mild cation exchange method was also reported for Mn-doped ZnSe QDs in the detection of Cd^2+^ and Hg^2+^ ions.^115^

**Fig. 7:**
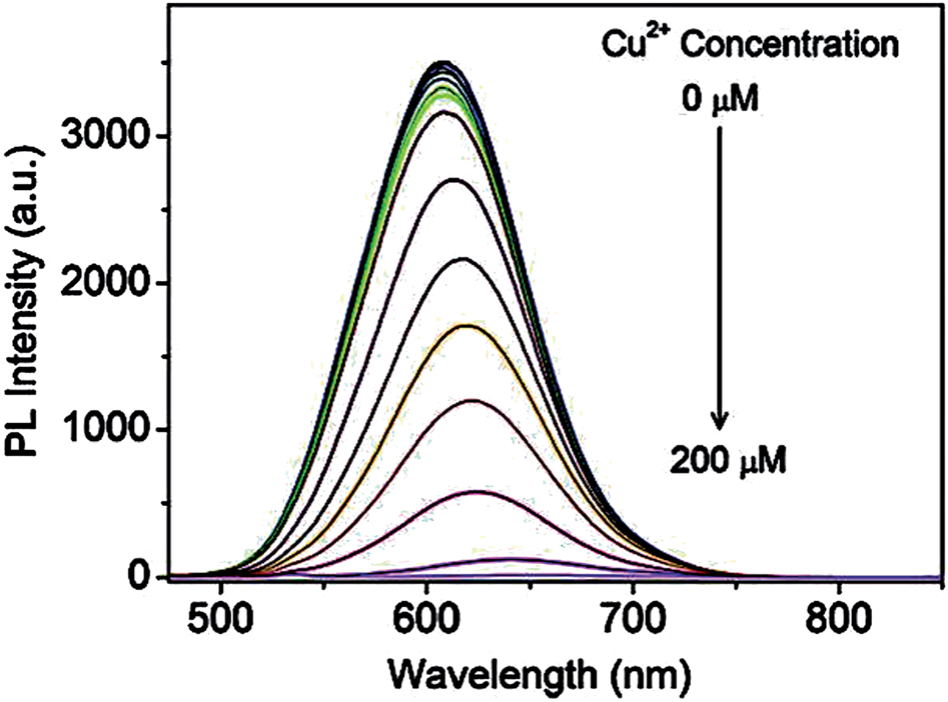
PL spectra of the CIZS/ZnS QDs in the presence of different amounts of Cu^2+^ ions [reprinted with permission from ref. 43 copyright 2019 Elsevier].

Recently, Jiao *et al.*^43^ reported GSH capped CuInZnS/ZnS (CIZS/ZnS) QD fluorescent probe for the detection of Cu^2+^ ions *via* cation exchange. The results showed PL quenching accompanied by red-shifted emission peaks at increased Cu^2+^ ions concentrations (Fig. 7).

The chemical compositions of QDs in the absence and presence of Cu^2+^ ions revealed a decrease in In and Zn content from 55.0% to 54.3% and 37.5% to 33.7% at increased Cu^2+^ concentrations, respectively, as shown in Table 2. Since ZnS has a wider bandgap than CuInS_2_, the replacement of Zn with Cu^2+^ resulted in a redshift of the absorbance and PL spectra. In addition, the cation-exchange of Zn and In in the QD core at different degrees occurs because although In is a harder Lewis acid than Zn, it is more difficult to exchange it with Cu^2+^ (weaker Lewis acid) due to In_2_S_3_ exhibiting a lower *k*_sp_ value than ZnS and Cu_2_S. Thus, In would exchange at a lower degree than Zn. PL lifetime studies displayed a decrease in lifetimes at increased Cu^2+^ ion concentrations from 413 ns to 289 ns (100 μm Cu^2+^ ions). This suggested a change in the recombination processes of the QDs, which was an indication of increased non-radiative surface defects, hence the decrease in the PL intensity.^43^

**Table 2:** Zinc and indium content in CIZS/ZnS QD in the presence of different amounts of Cu^2+^ ions.^43^

[Cu^2+^] detection (μM)	0	0.1	1	10	100
Cu content (%)	7.52	7.65	7.77	8.13	12
Zn content (%)	37.5	37.4	37.3	36.9	33.7
In content (%)	55.0	55.0	54.9	54.9	54.3

**Fig. 8:**
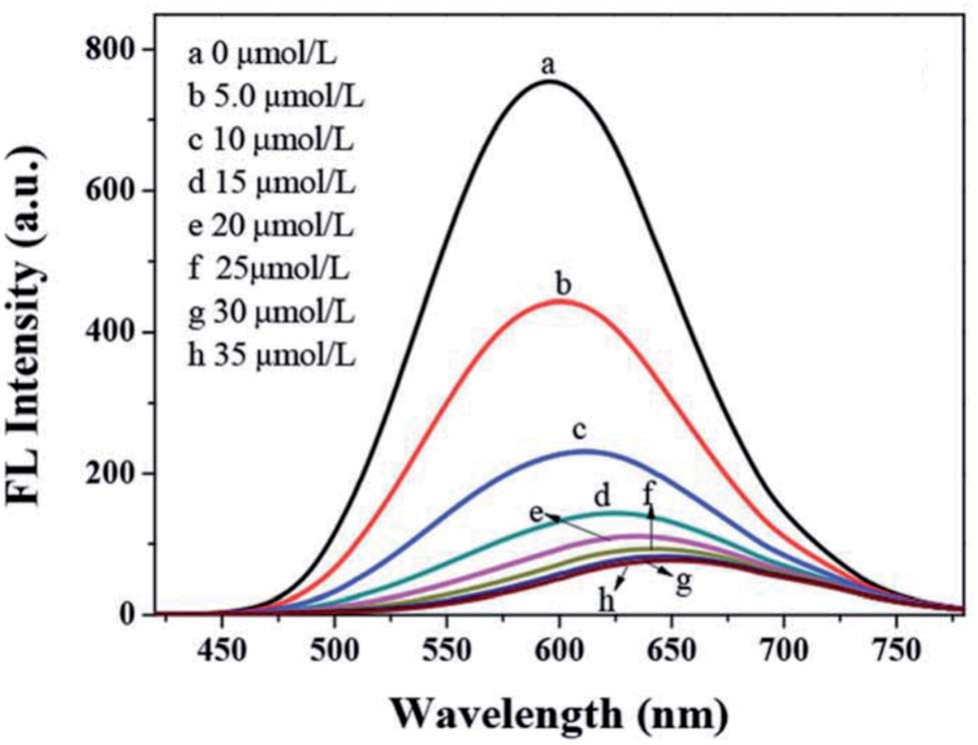
PL spectra quenching spectra of AIZS QDs with different concentrations of Cu^2+^ [reprinted with permission from ref. 91 Royal Society of Chemistry].

Han *et al.*^91^ displayed GSH capped AgInZnS for the detection of Cu^2+^ ions. The fluorescence intensity of the QDs also decreased with increase Cu^2+^ ion concentration with a gradual redshift in the emission peak (Fig. 8). These results suggest possible cation exchange interaction between Cu^2+^ ions and the cations in the QD core, as seen in other publications.^42,43^

**Fig. 9:**
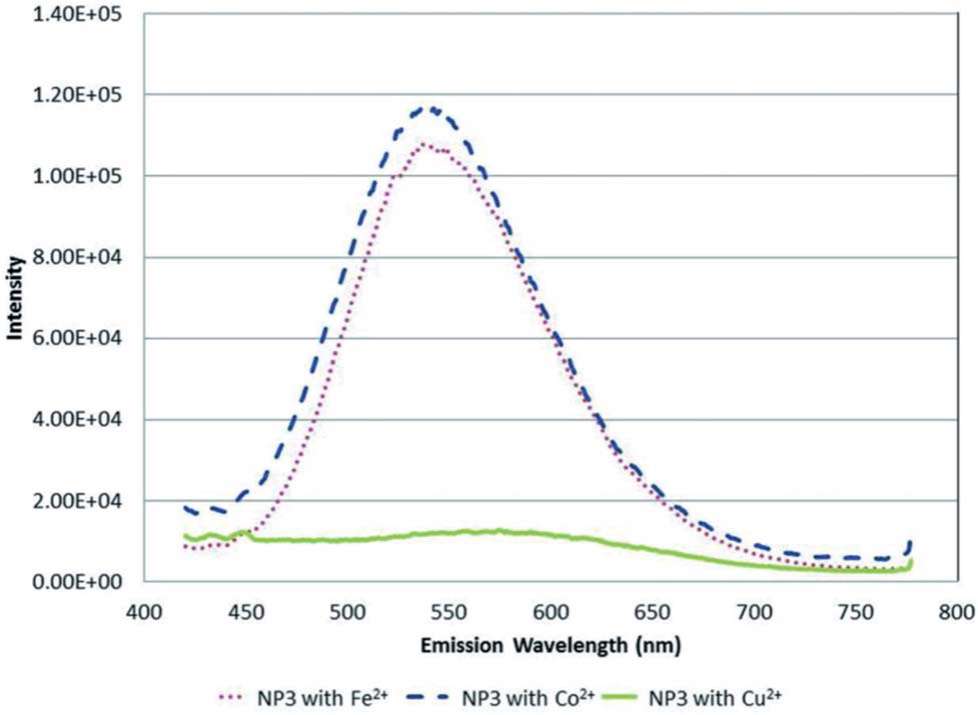
PL spectra of NP3 in CHCl_3_/CH_3_CN with 500 nM (∼1 ppm) of Fe^2+^, Co^2+^ and Cu^2+^ metal ions [reprinted with permission from ref. 89 American Chemical Society].

Cambrea *et al.*^89^ fabricated three AgInZnS NPs of different Zn : Ag : In mole ratios with identical non-specific ligand dodecylamine. The Zn : Ag : In mole ratios for NP1, NP2 and NP3 were 0 : 1 : 1, 0.6 : 0.7 : 0.7 and 1.2 : 0.4 : 0.4 respectively. The study focused on determining metal ion specificity detection based on the interaction of the metal ion with the core NP containing different metal mole ratios. The study showed a different response to all three NPs for each metal ion. For example, NP3 dissolved in CHCl_3_ and CH_3_CN was selective towards Cu^2+^ ions compared to Fe^2+^ and Co^2+^, as seen in the complete quenching of the PL peak at 500 nM (Fig. 9). In addition, the quenched PL peak maximum was seen at a longer wavelength indicating a redshift. On the other hand, NP1 showed complete quenching when exposed to dichromate while chromate had the least quenching effect even though both solutions were of chromium in the same valency state of +6. NP2 was significantly quenched by Hg^2+^ ions at 500 nM, whereas it was one of the least reactive analytes in NP1 and NP3. The report highlighted that most metal ions at mM ranges caused precipitates to form in quenched NP solution, which could be linked to fluorescence mechanism A (Fig. 2). However, since all metal ions responded differently to all the three NPs, the quenching mechanism might involve other interactions other than the cation exchange (fluorescence mechanism B), as shown in Fig. 2. Particularly, Cu^2+^ ion detection by NP3 could be partly attributed to cation exchange indicating direct interaction of the core NP with Cu^2+^ ions. These suspicions are based on the red-shift PL peak apart from the PL decrease also seen in the detection of the other metals. Since NP3 contains the highest amounts of Zn, it is most likely to experience cation exchange reactions between Zn and In in the QD and Cu^2+^ ions interaction than the other two NPs. However, further investigations are required to confirm this possibility.^89^

**Fig. 10:**
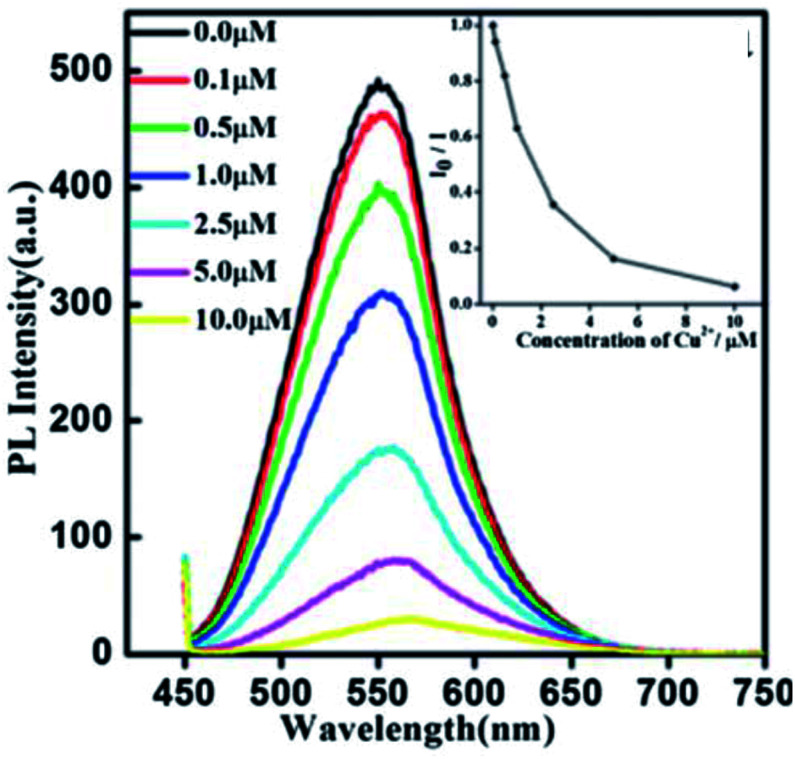
PL spectra of AgInS_2_/ZnS NCs (0.02 mg mL^−1^) exposed to different amounts of Cu^2+^. Inset: the correlation curve of *I*_0_/*I*_i_ as a function of the concentration of Cu^2+^ from 0.1 μM to 10 μM [reprinted with permission from ref. 42 Royal Society of Chemistry].

Xiong *et al.*^42^ reported the synthesis of GSH-capped AgInS_2_/ZnS core/shell QDs *via* microwave irradiation route. The core/shell material displayed quenched and red-shifted emission after the addition of Cu^2+^ ions (Fig. 10). This could be due to the replacement of Zn^2+^ with Cu^2+^ ions, forming a CuS layer on the QD surface, thus inducing the non-radiative recombinations.^42^ The red-shifted emission is not surprising since ZnS (3.54 eV) has a wider bandgap than CuS (1.97 eV).^116^

Several interactive routes that result in quenching may have been reported but a clear attribution of interactions remains a challenge, which leads to the suspicion that the interaction could be most likely a combination of interactive routes in some cases. For example, some authors reported the detection of Hg^2+^ using Cd based QDs through quenching *via* a combination of interaction mechanisms. The detection was attributed to both cation exchange between the QDs and the Hg^2+^ as well as ligand detachment from the QD surface.^112,113^ Future studies need to consider all possible pathways to obtain detailed information on the interactions involved in the detection process.

### 4.2 Fluorescence enhancement

Fluorescence enhancement is a process in which the fluorescence intensity of a fluorophore is enhanced due to its interaction with a molecule. This mechanism can occur as a result of the passivation of surface defects by either Zn^2+^ or Cd^2+^ ions.^62^ This is common in aqueous QDs because they normally have a lot of surface defects of S^2−^, Se^2−^, which result in low luminescence.^29^ Some authors have reported PL enhancement when QDs interacted with low concentrations of Ag^+^, while higher concentration induced PL quenching. As with Zn^2+^ and Cd^2+^, the Ag^+^ passivate surface defects but an excess will saturate traps and induce non-radiative recombination leading to PL quenching.^119,120^ Fluorescence enhancement is also affected by the size of the interacting QD; such that smaller sized QDs endure more surface defects than larger ones. Thus, smaller QDs may allow higher concentrations of the metal ion to passivate the surface while larger ones may require lower concentration resulting in varied detection ranges.^121^ The formation of Zn-complex and Ba-complex on the surface of the QD produced PL improvement. l-Cysteine capped CdS QDs formed a 3-dimensional network with Zn, which activated the surface state and improved the PL.^122^ This was also seen when Ba was added to mercaptoethanol-capped CdSe QDs.^123^

**Fig. 11:**
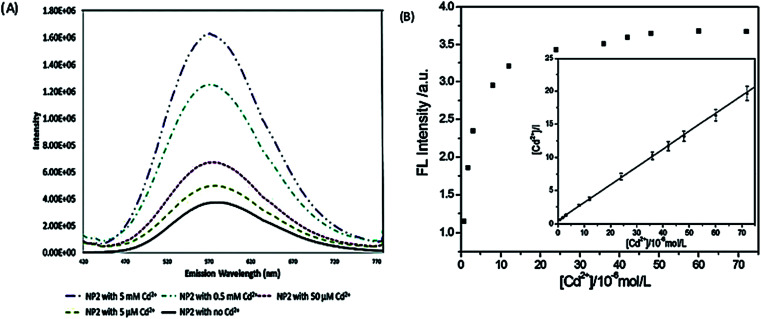
(A) PL spectra of NP2 in the presence of Cd^2+^ ions. As the amount of Cd^2+^ is increased the fluorescence increases by an order of magnitude [reprinted with permission from ref. 89 American Chemical Society]. (B) The relationship between the PL intensity of CuInS_2_ QDs and the concentration of Cd^2+^. Inset: the plot of [Cd^2+^]/I *versus* the concentration of Cd^2+^ [reprinted with permission from ref. 44 Royal Society of Chemistry].

**Fig. 12:**
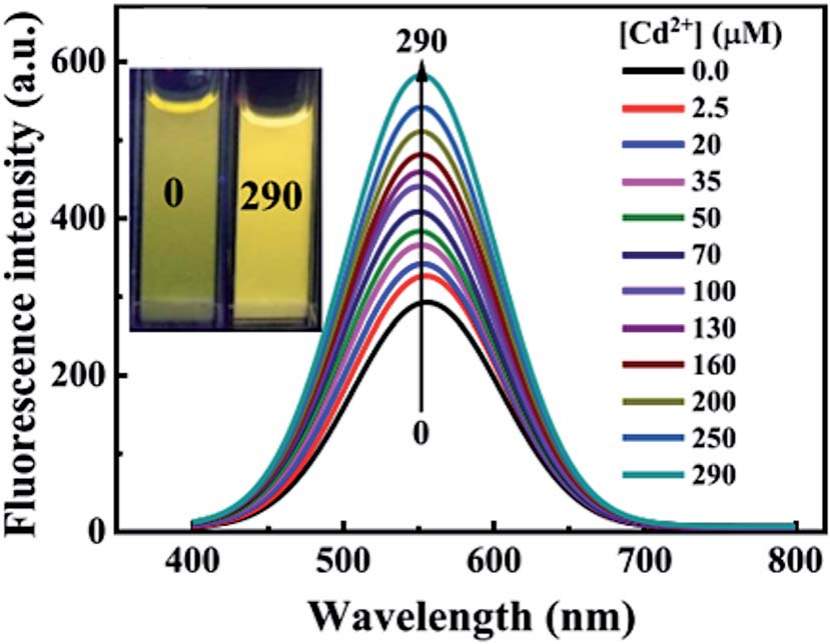
PL spectra of AgInZnS QDs with increased concentrations of Cd^2+^; inset shows images of AgInZnS QDs without (left) and with 290 μM Cd^2+^ under UV light irradiation [reprinted with permission from ref. 92 copyright 2021 Elsevier].

**Fig. 13:**
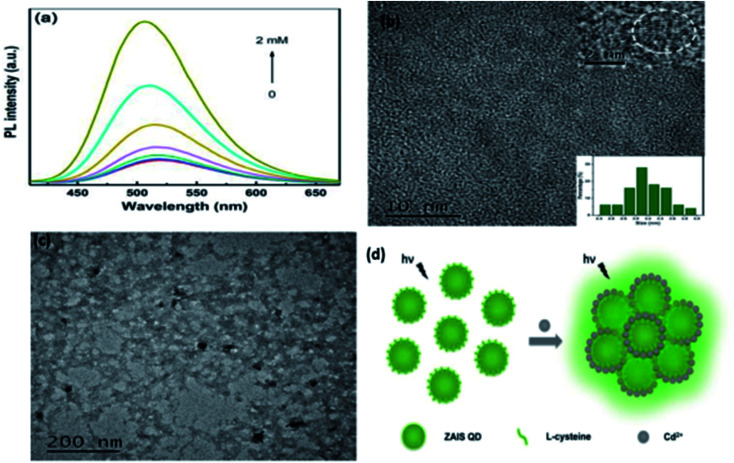
(a) PL spectra of Zn–Ag–In–S QDs without and with Cd^2+^ of different concentrations added (from 25 μM to 2 mM). (b) TEM image of ZAIS QDs. Inset: HRTEM image of the sample (top); size distribution histogram of QDs (bottom). (c) TEM image of ZAIS QDs after adding Cd^2+^ of 2 mM. (d) Illustration of possible mechanism of Cd^2+^-triggered AIEE of ZAIS QDs [reprinted with permission from ref. 93 Royal Society of Chemistry].

The study on the three AgInZnS NPs (of different metal ratios) for the detection of heavy metal ions revealed an increase in PL on exposure to Cd^2+^ ions for all the NPs (Fig. 11A).^89^ This is not surprising because core QDs are expected to have surface defects since they are bare. Thus, the addition of Cd^2+^ fills the defect sites and enhance the PL by reducing the non-radiative recombinations.^89^ Similar results were seen in MPA capped CuInS_2_ QDs (Fig. 11B),^44^ where the exposure to Cd^2+^ improved the PL intensity but PL quenching occurred at higher concentrations indicating saturation of the surface traps.^44^ MPA capped AgInZnS QDs (Fig. 12) also revealed continuous increase in PL intensity with increased Cd^2+^ concentrations due to surface defect passivation, which was verified by the prolonged fluorescence lifetimes and increased PLQY.^92^ The average lifetime and PLQY of the AgInZnS QDs increased from 262.55 ns to 292.33 ns and 41% to 49% respectively after the addition of 290 μM Cd^2+^. The AgInZnS QDs exhibited a zeta potential of −44 mV suggesting that Cd^2+^ ions adsorbed onto the negatively charged QD surface through electrostatic interactions, which passivated the surface trap state of the QDs. As a result, the PLQY was enhanced and the fluorescence lifetime were elongated.^92^ Recently, aggregation induced emission enhancement (AIEE) was seen in l-cysteine capped Zn–Ag–In–S quaternary QDs when exposed to Cd^2+^.^93^ The PL enhancement of the Zn–Ag–In–S QDs was accompanied by blue-shifted PL peaks and aggregation of the QDs (Fig. 13A). This was attributed to the binding of Cd^2+^ to thiol anions on the QD surface, leading to weakened electrostatic repulsion between the QDs and passivation of surface defects, TEM images (Fig. 13B and C) show the well dispersed Zn–Ag–In–S QDs and aggregated QDs when exposed Cd^2+.^ Furthermore, macroscopic floc formation was seen at high Cd^2+^ concentrations. The weakened electrostatic repulsion can be confirmed by the reduced negative charge after Cd^2+^ addition from −38.8 mV to −27.4 mV. The blue-shifted PL peaks at increased Cd^2+^ concentrations are suspected to be due to the diffusion of Cd^2+^ in the Zn–Ag–In–S QD broadening the band gap. The possible mechanism responsible for AIEE is shown in Fig. 13D.^93^

## 5. Selective detection of heavy metal ions

The QD surface functionality not only determines its solubility but also its PL properties. Different QDs exhibit different PL properties thus interacting differently and selectively to various heavy metal ions. It is these interactions that result in selective detection processes. Most interactions are based on direct and non-specific interaction which is the basis of many of the QD-probes discussed in this review. Table 3 shows a summary of ternary I III VI QDs based fluorescent probes for the detection of various heavy metal ions, which are elaborated in following sections.

**Table 3:** Summary of ternary I III VI QDs based fluorescent probes for the detection of heavy metal ions

QDs	Analyte	Detection scheme	Detection mechanism	Detection range	LOD	Reference	Sample type
MPA-capped CuInS_2_	Cu^2+^	PL quenching	Electron transfer	0.2–10 μM	0.10 μM	44	Tap and pound water
Cd^2+^ modified MPA-capped CuInS_2_	Cu^2+^	PL quenching	Electron transfer	0.1–10 μM	0.037 μM		
MPA-capped CuInS_2_	Cd^2+^	PL enhancement	Surface passivation	0.8–7.2 μM	0.19 μM		
DTAB-capped AgInZnS	Cu^2+^	PL quenching	Electron transfer	0.05–10 μM	15 nM	101	—
SDS-capped AgInZnS	Cu^2+^	PL quenching	Electron transfer	0–340 μM	27.3 nM	102	—
AgInZnS-GO	Cu^2+^	PL quenching	Binding with ligands	0–850 μM	0.18 nM	97	—
Optical fiber SDS capped AgInZnS	Cu^2+^	PL quenching	Electron transfer	2.45–800 nM	2.45 nM	103	Lake water
GSH-capped CuInZnS/ZnS	Cu^2+^	PL quenching	Cation-exchange	0.02–20 μM	6.7 nM	43	River water
GSH-capped AgInZnS	Cu^2+^	PL quenching	Cation-exchange	0–35 μM	100 nM	91	—
AgInZnS	Cu^2+^	PL quenching	Cation-exchange	Not mentioned	Not mentioned	89	—
AgInZnS	Hg^2+^	PL quenching	Cation-exchange	Not mentioned	Not mentioned		
AgInZnS	Cd^2+^	PL enhancement	Surface passivation	Not mentioned	Not mentioned		
AgInZnS	Cr^6+^	PL quenching	Cation-exchange	Not mentioned	Not mentioned		
GSH-capped AgInS_2_/ZnS	Cu^2+^	PL quenching	Cation-exchange	Not mentioned	Not mentioned	42	—
TGA-capped CuInS_2_/ZnS	Co^2+^	PL quenching	Inner filter effect	0.3012–90.36 μM	0.16 μM	90	—
MSA capped AgInS_2_	Pb^2+^	PL quenching	Not mentioned	0–90 nM	16.44 nM	57	—
GSH AgInS_2_–ZnS	Cr^3+^	PL quenching	Binding with ligands	0.025–10 μM	0.51 nM	100	—
MPA AgInZnS	Cd^2+^	PL enhancement	Surface passivation	0.1–290 μM	37.8 nM	92	Lake water
l-Cysteine Zn–Ag–In–S	Cd^2+^	PL enhancement	AIEE	25 μM to 2 mM	1.56 μM	93	Tap and lake water

### 5.1 Detection of Cu^2+^ ions

Copper is among the few heavy metals essential for human health. It becomes toxic at high concentrations, especially in water.^1^ A variety of surface-functionalized I–III–VI QDs has revealed selectivity for Cu^2+^ ions. MPA capped CuInS_2_ revealed electron transfer induced quenching interaction with Cu^2+^ ions. The electron transfer from the QD to Cu^2+^ facilitated the reduction of Cu^2+^ to Cu^+^, which resulted in Cu_2_S precipitate on the surface. A good linear relationship between the QDs and Cu^2+^ concentrations was achieved at a range of 0.2–10 μM with an LOD of 0.10 μM as shown in Table 3. Detection equilibrium was reached within 15 minutes and at an optimum pH of 7.4. The detection range and LOD were improved to 0.1–10 μM and 0.037 μM respectively by using Cd^2+^ modified CuInS_2_ QDs; which allowed the detection of Cu^2+^ in the presence of Cd^2+^ ions. In this probe, the Cu^2+^ ions adsorbed on the QD surface changing the CuInS_2_–Cd-SR orientation thus inducing non-radiative recombinations which lead to quenching. Physiological ions had negligible interference while some interference was seen in the detection of Hg^2+^ and Pb^2+^, which could be resolved by complexing with NH_3_F and thiosemicarbazide respectively. The probes were tested for tap and pond water and recoveries of 95.6–108% and 93.8–103.4% were achieved respectively.^44^

Zinc doped AgInS_2_ (AgInZnS) QDs synthesized *via* an emulsion solvent route with DTAB as amphiphilic ligand were reported by Liu *et al.*^107^ XPS analysis confirmed the formation of the QDs by identifying the valences of Ag, In, Zn, and S to be 1+, 3+, 2+, and 2−. However, Cu (2p) peak corresponding to Cu^+^ was also identified when the QDs were exposed to Cu^2+^ ions. This confirmed the electron-induced PL quenching resulting in the reduction of Cu^2+^ to Cu^+^. A detection range of 0.05–10 μM and LOD of 15 nM were achieved. The probe displayed a rapid response time of one minute and a sequence of QDs, Tris–HCl buffer, and Cu^2+^ ions yielded the best results. No further quenching occurred at a concentration higher than 10 μM indicating saturated surface traps. Other metal ions had no significant influence on the PL intensity but Fe^3+^ interference was eliminated by adding FeF_6_^3−^.^107^

Liu *et al.*^108^ displayed a wider detection range of 0–340 μM with SDS capped AgInZnS QDs at longer response times of 10 minutes. The importance of the probe concentration was stressed since a high concentration might allow little analyte to interact compromising sensitivity while a low concentration would offer limited sites to adsorb. As a result, an optimum concentration of 0.5 mg mL^−1^ was established. The reported zeta potential value of −36.8 mV suggested an electrostatic interaction between the QD and Cu^2+^ existed, aiding the electron transfer from QDs to Cu^2+^ ions. Additionally, the use of different Cu^2+^ ion sources (*i.e.* CuCl_2_, CuN0_3_) did not affect the PL intensity at the same concentration (340 μM).^108^

A wider detection range of 0–850 μM was achieved with AgInZnS–graphene oxide nanocomposite with a response time of one minute.^99^ The AgInZnS–GO–Cu^2+^ interaction triggered particle size increase due to the R–COO–Cu^2+^–COOR complex formation, which quenched the PL of the NC. This result was seen in the obvious red-shifted emission after the addition of Cu^2+^, while also confirmed by TEM images illustrating a size of 5.7 nm and 21.7 nm before and after the addition of Cu^2+^ ions respectively. The NC revealed weak binding interactions with other metal ions because only slight changes in PL intensity were seen.^99^

The detection of Cu^2+^ at the nanomolar range was later reported by Liu *et al.*,^109^ using a fluorometric optical fiber nanoprobe based on SDS-capped AgInZnS QDs. A detection range of 2.45–800 nM and LOD of 2.45 nM were reported. Electron transfer was put forward as a possible mechanism. PL lifetime studies supported the possibility where average lifetimes decreased from 500 ns to 325 ns for QDs in the presence and absence of Cu^2+^ respectively. Furthermore, the detection could be carried out in the presence of Fe^3+^ by adding FeF_6_^3−^. Applications in real water samples produced recoveries between 95.7–100.5%.^109^

A few authors revealed GSH capped QD based fluorescent probes towards the detection of Cu^2+^ ions through cation exchange mechanism.^42,43,91^ CuInZnS/ZnS core/shell QDs synthesized with a 1/1 Cu/Zn mole ratio exhibited an emission peak at 607 nm. The emission intensity decreased while red-shifted emission from 607 nm to 646 nm was observed with increased Cu^2+^ ion concentration. The probe claimed detection between 0.02–20 μM with an LOD of 6.7 nM. Other metal ions (of the same concentration as Cu^2+^) had no significant change upon their addition and no emission shifts were observed. GSH offers a variety of functional groups on the surface that helps resist pH variation and maintain QD stability. As a result, the probe responded to wide pH ranges (6–10). The strategy was applied in real water samples. Recoveries between 98.07–102.4% were achieved, which were comparable to ICP-AES results of the same sample.^43^ AgInZnS QDs were quenched with slight red-shifted emission when exposed to Cu^2+^ ions. The QDs displayed a good linear relationship between quenching efficiency and Cu^2+^ ion concentration, thus a detection range and LOD of 0–35 μM and 100 nM were established respectively.^91^ In another development, AgInS_2_/ZnS core/shell QDs experienced the replacement of Zn^2+^ with Cu^2+^ forming a CuS layer on the QD surface. This detection scheme was supported by the gradual quenching and red-shifted emission between 0.1–10 μM.^42^

AgInZnS NPs synthesized with different metal mole ratios (1 = no Zn, 2 = low Zn, 3 = high Zn) were applied to Cu^2+^ detection.^89^ A direct interaction was assumed between the QDs and Cu^2+^ resulting to a significant quenching and red-shifted emission with the NP at Zn concentrations as low as 500 nM. The results suggested a cation-exchange mechanism between the Zn and In in the NP and Cu^2+^ ions.^89^

### 5.2 Detection of other heavy metal ions

Most studies focused on Cu^2+^ ions^42–44,89,91,99,104,105^ but some authors also reported potential probes for other metal ions such as Cd^2+^, Hg^2+^, Cr^6+^, Pb^2+^, and Co^2+^.^44,57,89,90^ In the previously discussed study by Cambrea *et al.*,^89^ the detection of Cd^2+^ ions were also explored. The results demonstrated a passivating effect on the AgInZnS NPs. For example, NP2 (Zn : Ag: In mole ratios of 0.6 : 0.7 : 0.7) showed a gradual increase in PL intensity between 5 μM and 5 mM.^89^ The passivating effect was also seen in Liu *et al.*^44^ The Langmuir model was used to describe the relationship between the PL intensity of QDs and Cd^2+^ concentration. A linear relationship was seen at 0.8–7.2 μM with an LOD of 0.19 μM. Zn is also known to have a passivating effect on QDs. However, Cd^2+^ has a higher affinity for thiol group (*S*–*R*) than Zn^2+^. This could be confirmed in the 3.6-fold PL intensity increase when Cd^2+^ was added while a decrease was seen when Zn^2+^ was added at a similar concentration. In addition, physiological metal ions had little effect while Pb and Hg had some effect on the PL intensity of the QDs, which were eliminated by complexing with NH_3_F and thiosemicarbazide.^44^ MPA capped AgInZnS QDs were employed as Cd^2+^ detectors in aqueous solutions based on adsorption of Cd^2+^ through an electrostatic interaction between Cd^2+^ ions and the negatively charged QD surface, resulting in enhanced PLQY and elongated fluorescence lifetimes.^92^ The QD probes exhibited a linear relationship between PL intensity and Cd^2+^ concentration in the 0.1–290 μM range with a LOD of 37.8 nM. Moreover, the detection of Cd^2+^ in lake water was demonstrated, with % recoveries between 96% and 102%, which was comparable to those found using ICP-AES.^92^l-Cysteine capped Zn–Ag–In–S QDs exhibited Cd^2+^ detection in aqueous solution based on AIEE.^93^ The QDs showed linear PL intensity enhancement between 25 μM and 2 mM with a LOD of 1.56 μM and could be applied in tap and pond water samples with recoveries between 98.1% and 103.3%. The effect of common metal cations on the PL response of the QDs was investigated. The results showed little effect towards PL intensity of the QDs after addition of Ag^+^, Ba^2+^, Fe^3+^, K^+^, Mg^2+^, Mn^2+^, Na^+^ and Zn^2+^ while Cd^2+^ resulted in three times enhanced PL intensity. On the other hand Pb^2+^, Hg^2+^ and Cu^2+^ quenched the PL intensity, which can be overcome by complexing Pb^2+^ and Hg^2+^ with ammonium fluoride and thiosemicarbazide^44^ and Cu^2+^ with l-cysteine.^124^ Thus, complexing Cu^2+^ with l-cysteine prior detection resulted in reduced degree of quenching, demonstrating a possible PL enhancement of the QDs in the presence of common cations.^93^

In the case of Hg^2+^ ions, a significant reduction in the PL intensity of AgInZnS NPs with low Zn (NP2) was seen at 500 nM, which was the opposite of NPs with high (NP3) and no Zn (NP1). This means greater interaction occurred between NP2 and Hg than the other two NPs. On the other hand, NP1 showed complete quenching when exposed to 500 nM dichromate and little quenching with chromate at the same concentration, suggesting that ion size or overall charge may influence selectivity.^89^

In another development, GSH capped AgInS_2_/ZnS QDs displayed PL quenching and red-shifted emission when exposed to increased concentrations of Cr^3+^, which was attributed to Cr^3+^–GSH complex formation on the QD surface resulting in aggregation.^100^ A linear relationship between quenching efficiency and Cr^3+^ concentration was seen between 0.025 μM and 10 μM with a LOD of 0.51 nM. The effect of different metal cations across the periodic table towards the PL response of the QDs was investigated. The studies revealed almost 85% of PL quenching of the QDs and lower degree of quenching for Pb^2+^, Cu^2+^, Ni^2+^ and Hg^2+^, while other metal ions exhibited negligible effect on the PL intensity of the QDs. Cr^3+^ ions were selectively detected by adding Na_2_S to mask the interfering metal ions (*i.e.* Pb^2+^, Cu^2+^, Ni^2+^ and Hg^2+^) which have a greater affinity to sulfides. Results showed that the addition of the Na_2_S prevented the PL quenching of the QDs in the presence of the interfering metal ions, leaving the quenching due to Cr^3+^ unaffected. In addition, Cr^3+^ were selectively detected by the QDs among a mixture of the interfering metal ions.^100^

The interaction of Co^2+^ ions with TGA capped CuInS_2_/ZnS QDs resulted in the formation of a TGA–Co^2+^–TGA due to the detachment of the TGA molecule on the QDs, leading to quenching of QDs.^100^ The quenching was partly attributed to IFE. A good linear relationship between quenching efficiency and Co^2+^ concentration was seen between the range of 0.3012–90.36 μM with an LOD of 0.16 μM.^100^ Furthermore, mercaptosuccinic acid (MSA) capped AgInS_2_ core QDs showed potential in the detection of Pb^2+^ ions. The addition of Cd^2+^, Zn^2+^, K^+,^ and Mn^2+^ at increased concentrations had little effect on PL intensity of QDs but Pb^2+^ produced a gradual decrease at the same concentrations. A good linear relationship between PL efficiency and Pb^2+^ ion concentration was seen between 0 – 90 nM with a detection limit of 16.44 nM.^57^

## 6. Conclusions and future work

We have reviewed the progressive research on I–III–VI ternary QDs for fluorescence detection of heavy metal ions such as Cu^2+^, Hg^2+^, Cr^2+^, Co^2+^, Pb^2+^, and Cd^2+^ in water. These materials were found to be safer alternatives compared to the traditional QDs for QD-based detectors. A large fraction of reviewed reports focused on the Cu^2+^ ion detection and only a few reports were available on other metal ions. Most detection mechanisms put forward were based on the interaction of the host QDs and the metal ion rather than ligand specific-functionalized QDs which have been reported to exhibit improved selectivity. Nevertheless, I–III–VI QDs have demonstrated potential for applications in the treatment of environmental water samples. Although interference studies aimed to improve selectivity (*i.e.* complexing interfering ions and modifying the probe with the interfering ions), most interference studies focused on investigating the influence of other metal ions separately. It would be interesting to see interference studies that illustrate a better representation of a real water environment *i.e.* investigating interferences of metal ions as a collective rather than separately, to give more insight on the applicability of these methods in real life. Studies on the interference of other metal ions at higher concentrations than the target ion may also bring more insight. Among the various fluorescent nanomaterials, ternary I–III–VI QDs have emerged as a new class of non-toxic nanomaterials with excellent fluorescent sensing abilities for heavy metals. They exhibit small sizes that could potentially present a high surface area, yet they are rarely utilized in heavy metal ion removal studies. Their ability to detect heavy metals, which is mostly attributed to the binding of the metals to the surface functional groups is a phenomenon which describes one of the many ways that adsorption occurs. Thus, future applications could involve exploring I–III–VI QDs and their composites for water remediation studies in addition to the currently exploited detection studies. Furthermore, their promising applications as heavy metal probes could expand their use as cellular probes for tracing metal ions in living systems.

## Conflicts of interest

The authors declare that there are no conflicts of interest.

## Supplementary Material

## References

[cit1] Jaishankar M., Tseten T., Anbalagan N., Mathew B., Beeregowda K. (2014). Toxicol.

[cit2] Xie W., Peng C., Wang H., Chen W. (2017). Int. Res. J. Publ. Environ. Health.

[cit3] Environmental Chemistry for a Sustainable World, ed. S. Stankovic, M. Jovic, A. R. Stankovic, L. Katsikas E. Lichtfouse, J. Schwarzbauer, D. Robert, Springer, Dordrecht, 2014, pp. 313–362

[cit4] Sarioz O., Surme Y., Muradoglu V. (2013). Chem. Pap..

[cit5] Xiang Y., Lu Y. (2013). Chem. Commun..

[cit6] Addo-Bediako A., Matlou K., Makushu E. (2018). J. Aquat. Sci..

[cit7] Bader N. R. (2011). Rasayan J. Chem..

[cit8] Inui T., Kosuge A., Ohbuchi A., Fujita K., Koike Y., Kitano M. (2012). Am. J. Anal. Chem..

[cit9] Fu L., Zhuang J., Lai W., Que X., Lu M., Tang D. (2013). J. Mater. Chem. B.

[cit10] Qiu Z., Shu J., Jin G., Xu M., Wei Q., Chen G., Tang D. (2016). Biosens. Bioelectron..

[cit11] Qiu Z., Tang D., Shu J., Chen G., Tang D. (2016). Biosens. Bioelectron..

[cit12] Chen J., Tang J., Zhou J., Zhang L., Chen G., Tang D. (2014). Anal. Chim. Acta.

[cit13] Zhuang J., Fu L., Tang D., Xu M., Chen G., Yang H. (2013). Biosens. Bioelectron..

[cit14] Zhou Q., Lin Yo, Lin Yu, Wei Q., Chen G., Tang D. (2016). Biosens. Bioelectron..

[cit15] Zhang B., Chen J., Liu B., Tang D. (2015). Biosens. Bioelectron..

[cit16] Zhuang J., Fu L., Xu M., Zhou Q., Chen G., Tang D. (2013). Biosens. Bioelectron..

[cit17] Ke J. , Semiconductor Nanocrystal-Based Nanosensors and Metal Ions Sensing, Elsevier Inc, 2020, ch. 3, pp. 79–117

[cit18] Wu P., Zhao T., Hou X. (2014). Nanoscale.

[cit19] De Acha N., Elosúa C., Corres J. M., Arregui F. J. (2019). Sensors.

[cit20] Chandan H. R., Schiffman J. D., Balakrishna R. G. (2018). Sens. Actuators, B.

[cit21] Guo Y., Zhang L., Zhang S., Yang Y., Chen X., Zhang M. (2015). Biosens. Bioelectron..

[cit22] Bronzato J. D., Tofanello A., Oliveira M. T., Bettini J., Brito A. M. M., Costa S. A., Costa S. M., Lanfredi A. J. C., Nascimento O. R., Nantes-Cardoso I. L. (2022). Appl. Surf. Sci..

[cit23] You J., Guo Y., Guo R., Liu X. (2019). Chem. Eng. Sci..

[cit24] Reza Ganjali M., Al-Naqshabandi M. A., Larijani B., Badieif A., Vatanpour V., Reza Rajabi H., Rezaniag H., Pazireshg S., Mahmodii G., Kimi S.-J., Reza Saeb M. (2021). Chem. Eng. Res. Des..

[cit25] May B. M. M., Parani S., Oluwafemi O. S. (2019). Mater. Lett..

[cit26] Lakowicz J. R. , Fluorescence Sensing, in, Principles of Fluorescence Spectroscopy, J. R. Lakowicz, Springer, Boston, MA, 2006, pp. 623–673

[cit27] Reza Rajabi H., Shahrezaei F., Farsi M. (2016). J. Mater. Sci.: Mater. Electron..

[cit28] Adegoke O. , Dabrowski J. M., Montaseri H., Nsibande S. A., Petersen F., Republic of South Africa, 2017, pp. 1–222

[cit29] Elfeky S. A. (2018). J. Environ. Anal. Chem..

[cit30] Pu C., Qin H., Gao Y., Zhou J., Wang P., Peng X. (2017). J. Am. Chem. Soc..

[cit31] Jaiswal A., Ghsoh S. S., Chattopadhyay A. (2012). Langmuir.

[cit32] Xie R., Rutherford M., Peng X. (2009). J. Am. Chem. Soc..

[cit33] Hauck T. S., Anderson R. E., Fischer H. C., Newbigging S., Chan W. C. W. (2010). Small.

[cit34] Kolny-Olesiak J., Weller H. (2013). ACS Appl. Mater. Interfaces.

[cit35] Tsolekile N., Parani S., Matoetoe M. C., Songca S. P., Oluwafemi O. S. (2017). Nano-Struct. Nano-Objects.

[cit36] Girma W. M., Fahmi M. Z., Permadi A., Abate M. A., Chang J.-Y. (2017). J. Mater. Chem. B.

[cit37] Liu S., Su X. (2014). RSC Adv..

[cit38] Yarema O., Yarema M., Wood V. (2018). Chem. Mater..

[cit39] Zhu C., Chen Z., Gao S., Goh B. L., Samsudin I. B., Lwe K. W., Wu Y., Wu C., Su X. (2019). Prog. Nat. Sci..

[cit40] Wang L., Guan Z., Tang A. (2020). J. Nanopart. Res..

[cit41] Sandroni M., Wegner K. D., Aldakov D., Reiss P. (2017). ACS Energy Lett..

[cit42] Xiong W. W., Yang G. H., Wu X. C., Zhu J. J. (2013). J. Mater. Chem. B.

[cit43] Jiao M., Li Y., Jia Y., Yang Z., Luo X. (2019). Sens. Actuators, B.

[cit44] Liu S., Li Y., Su X. (2012). Anal. Methods.

[cit45] Kim S., Kang M., Kim S., Heo J.-H., Noh J. H., Hyuk Im S., Il Seok S., Kim S.-W. (2013). ACS Nano.

[cit46] Valizadeh A., Mikaeli H., Samiei M., Farkhani S. M., Zarghami N., Kouhi M., Akbarzadeh A., Davaran S. (2012). Nanoscale Res. Lett..

[cit47] Mao B., Chuang C. H., McCleese C., Zhu J., Burda C. (2014). J. Phys. Chem. C.

[cit48] Firoozi N., Dehghani H., Afrooz M. (2015). J. Power Sources.

[cit49] Li P. N., Ghule A. V., Chang J. Y. (2017). J. Power Sources.

[cit50] Choi H., Jeong S. (2018). Int. J. Precis. Eng. Manuf. - Green Technol..

[cit51] Wang G., Wei H., Shi J., Xu Y., Wu H., Luo Y., Li D., Meng Q. (2017). Nano Energy.

[cit52] Abate M. A., Chang J. Y. (2018). Sol. Energy Mater. Sol. Cells.

[cit53] Mao B., Chuang C. H., Wang J., Burda C. (2011). J. Phys. Chem. C.

[cit54] Bai X., Purcell-Milton F., Gun’ko Y. K. (2019). Nanomaterials.

[cit55] Chevallier T., Le Blevennec G., Chandezon F. (2016). Nanoscale.

[cit56] Sharma D. K., Hirata S., Bujak L., Biju V., Kameyama T., Kishi M., Torimoto T., Vacha M. (2017). Phys. Chem. Chem. Phys..

[cit57] Chen Y., Wang Q., Zha T., Min J., Gao J., Zhou C., Li J., Zhao M., Li S. (2018). J. Alloys Compd..

[cit58] Chetty S. S., Praneetha S., Vadivel Murugan A., Govarthanan K., Verma R. S. (2020). ACS Appl. Mater. Interfaces.

[cit59] Kang X., Yang Y., Huang L., Tao Y., Wang L., Pan D. (2015). Green Chem..

[cit60] Komarala V. K., Xie C., Wang Y., Xu J., Xiao M. (2012). J. Appl. Phys..

[cit61] Pons T., Pic E., Lequeux N., Cassette E., Bezdetnaya L., Guillemin F., Marchal F., Dubertret B. (2010). ACS Nano.

[cit62] Li L., Pandey A., Werder D. J., Khanal B. P., Pietryga J. M., Klimov V. I. (2011). J. Am. Chem. Soc..

[cit63] Fu M., Luan W., Tu S. T., Mleczko L. (2015). J. Nanomater..

[cit64] Yarema O., Yarema M., Bozyigit D., Lin W. M. M., Wood V. (2015). ACS Nano.

[cit65] Li L., Reiss P., Protie M. (2009). Small.

[cit66] Song W. S., Kim J. H., Yang H. (2013). Mater. Lett..

[cit67] Song W. S., Jang E. P., Kim J. H., Jang H. S., Yang H. (2013). J. Nanoparticle Res..

[cit68] Rao P., Yao W., Li Z., Kong L., Zhang W., Li L. (2015). Chem. Commun..

[cit69] Zhou R., Sun S., Li C., Wu L., Hou X., Wu P. (2018). ACS Appl. Mater. Interfaces.

[cit70] Ding K., Jing L., Liu C., Hou Y., Gao M. (2014). Biomaterials.

[cit71] Sitbon G., Bouccara S., Tasso M., Francois A., Bezdetnaya L., Marchal F., Beaumont M., Pons T. (2014). Nanoscale.

[cit72] Wang S., Jarrett B. R., Kauzlarich S. M., Louie A. Y. (2007). J. Am. Chem. Soc..

[cit73] Guo W., Chen N., Tu Y., Dong C., Zhang B., Hu C., Chang J. (2013). Theranostics.

[cit74] Chang J. Y., Chen G. R., Li J. D. (2016). Phys. Chem. Chem. Phys..

[cit75] Cheng C. Y., Ou K. L., Huang W. T., Chen J. K., Chang J. Y., Yang C. H. (2013). ACS Appl. Mater. Interfaces.

[cit76] Castro S. L., Bailey S. G., Raffaelle R. P., Banger K. K., Hepp A. F. (2004). J. Phys. Chem. B.

[cit77] Bensebaa F., Durand C., Aouadou A., Scoles L., Du X., Wang D., Le Page Y. (2010). J. Nanoparticle Res..

[cit78] Liu S., Zhang H., Qiao Y., Su X. (2012). RSC Adv..

[cit79] Che D., Zhu X., Wang H., Duan Y., Zhang Q., Li Y. (2016). J. Colloid Interface Sci..

[cit80] Malik M. A., O'Brien P., Revaprasadu N. (1999). Adv. Mater..

[cit81] Langevin M. A., Pons T., Ritcey A. M., Allen C. N. ì. (2015). Nanoscale Res. Lett..

[cit82] Xiang W., Xie C., Wang J., Zhong J., Liang X., Yang H., Luo L., Chen Z. (2014). J. Alloys Compd..

[cit83] Xie R., Kolb U., Li J., Basché T., Mews A. (2005). J. Am. Chem. Soc..

[cit84] Dubois F., Mahler B., Dubertret B., Doris E., Mioskowski C. (2007). J. Am. Chem. Soc..

[cit85] Feng J., Sun M., Yang F., Yang X. (2011). Chem. Commun..

[cit86] Zhang W., Zhong X. (2011). Inorg. Chem..

[cit87] Zhang J., Xie R., Yang W. (2011). Chem. Mater..

[cit88] Chen Y., Li S., Huang L., Pan D. (2013). Inorg. Chem..

[cit89] Cambrea L. R., Yelton C. A., Meylemans H. A. (2015). Trace Mater. Air, Soil, Water.

[cit90] Zi L., Huang Y., Yan Z., Liao S. (2014). J. Lumin..

[cit91] Han X. L., Li Q., Hao H., Liu C., Li R., Yu F., Lei J., Jiang Q., Liu Y., Hu J. (2020). RSC Adv..

[cit92] Liu Y., Tang X., Deng M., Zhu T., Edman L., Wang J. (2021). J. Alloys Compd..

[cit93] Wei C., Wei X., Hu Z., Yang D., Mei S., Zhang G., Su D., Zhang W., Guo R. (2019). Anal. Methods.

[cit94] Lakowicz J. R. , Quenching of Fluorescence in, Principles of Fluorescence Spectroscopy, J. R. Lakowicz, Springer, Boston, MA, 2006, pp. 237–265

[cit95] Qiu Z., Shu J., Tang D. (2017). Anal. Chem..

[cit96] Shu J., Tang D. (2017). Chem. - Asian J..

[cit97] Lin Z., Lv S., Zhang K., Tang D. (2017). J. Mater. Chem. B.

[cit98] Qiu Z., Shu J., He Y., Lin Z., Zhang K., Lv S., Tang D. (2017). Biosens. Bioelectron..

[cit99] Liu Y., Deng M., Tang X., Zhu T. (2016). J. Phys.: Conf. Ser..

[cit100] Parani S., Oluwafemi O. S. (2020). Nanotechnology.

[cit101] Omary M. A. , Patterson H. H., Luminescence, theory. in Encyclopedia of Spectroscopy and Spectrometry, 2016, Elsevier Ltd, pp. 636–653

[cit102] Badarau A., Dennison C. (2011). J. Am. Chem. Soc..

[cit103] Dong Y., Wang R., Li G., Chen C., Chi Y., Chen G. (2012). Anal. Chem..

[cit104] Zheng M., Xie Z., Qu D., Li D., Du P., Jing X. B., Sun Z. (2013). ACS Appl. Mater. Interfaces.

[cit105] Anand V. D., Deshmukh G. S., Pandey C. M. (1961). Anal. Chem..

[cit106] Gore A. H., Gunjal D. B., Kokate M. R., Sudarsan V., Anbhule P. V., Patil S. R., Kolekar G. B. (2012). ACS Appl. Mater. Interfaces.

[cit107] Liu Y., Deng M., Zhu T., Tang X., Han S., Huang W., Shi Y., Liu A. (2017). J. Lumin..

[cit108] Liu Y., Zhu T., Deng M., Tang X., Han S., Liu A., Bai Y., Qu D., Huang X., Qiu F. (2018). J. Lumin..

[cit109] Liu Y., Tang X., Huang W., Yin G., Deng M., Cao Y., Shi L., Zhu T., Huang L., Ikechukwu I. P., Gong Y., Bai Y., Qu D., Huang X., Qiu F. (2020). Microchim. Acta.

[cit110] Huang L., Wang J., Wang Q., Tang D., Lin Y. (2020). Microchim. Acta.

[cit111] Zhu L., Lv Z., Yin Z., Tang D. (2021). Anal. Chim. Acta.

[cit112] Lin Y., Zhou Q., Tang D., Niessner R., Yang H., Knopp D. (2016). Anal. Chem..

[cit113] Rivest J. B., Jain P. K. (2013). Chem. Soc. Rev..

[cit114] Leung L. K., Komplin N. J., Ellis A. B., Tabatabaie N. (1991). J. Phys. Chem..

[cit115] Zhou Z. Q., Yang L. Y., Yan R., Zhao J., Liu Y. Q., Lai L., Jiang F. L., Maskow T., Liu Y. (2017). Nanoscale.

[cit116] Tanveer M., Cao C., Ali Z., Aslam I., Idrees F., Khan W. S., But F. K., Tahir M., Mahmood N. (2014). CrystEngComm.

[cit117] Zhu X., Zhao Z., Chi X., Gao J. (2013). Analyst.

[cit118] Yuan C., Zhang K., Zhang Z., Wang S. (2012). Anal. Chem..

[cit119] Chen J. L., Zhu C. Q. (2005). Anal. Chim. Acta.

[cit120] Sahu A., Kang M. S., Kompch A., Notthoff C., Wills A. W., Deng D., Winterer M., Frisbie C. D., Norris D. J. (2012). Nano Lett..

[cit121] Xia Y. S., Cao C., Zhu C. Q. (2008). J. Lumin..

[cit122] Chen Y., Rosenzweig Z. (2002). Anal. Chem..

[cit123] Mahmoud W. E. (2012). Sens. Actuators, B.

[cit124] Huang P., Li S., Gao N., Wu F. (2015). Analyst.

